# Degradation of Paracetamol and Its Oxidation Products in Surface Water by Electrochemical Oxidation

**DOI:** 10.1089/ees.2018.0023

**Published:** 2018-11-05

**Authors:** Miguel Ángel López Zavala, Camila Renee Jaber Lara

**Affiliations:** Tecnológico de Monterrey, Water Center for Latin America and the Caribbean, Monterrey, Nuevo León, Mexico.

**Keywords:** active chlorine species, direct current (DC), electrochemical oxidation, oxidation products, paracetamol, surface water

## Abstract

Paracetamol and its toxic transformation products have been found in surface water, wastewater, and drinking water. Effective methods to degrade these products must be found to reduce their detrimental effects on microorganisms in aquatic systems and minimize the concern on human health. Thus, this study looked into the electrochemical oxidation of paracetamol and its oxidation products on surface water, and results were compared with those of paracetamol synthetic solution oxidation. Degradation of paracetamol was conducted using a stainless steel electrode cell, a pH of 3, and direct current densities of 5.7 mA/cm^2^ (6 V) and 7.6 mA/cm^2^ (12 V). For both current densities applied, the pharmaceutical and its oxidation products observed by high-performance liquid chromatography with diode-array detection (HPLC-DAD) at 254 nm were totally degraded. Faster degradation of paracetamol was observed at a higher current density. Indeed, 95% of paracetamol was oxidized in only 15 min at the 7.6 mA/cm^2^ current density. In comparison to the paracetamol synthetic solution's oxidation, degradation of paracetamol was faster in the surface water than the synthetic solution, at 5.7 mA/cm^2^. Nevertheless, at 7.6 mA/cm^2^, total degradation of paracetamol in surface water was delayed up to 40 min, versus 7.5 min in the synthetic solution. Three oxidation products, observed by HPLC-DAD at 254 nm, were fully oxidized. In comparison with the paracetamol synthetic solution, degradation of the oxidation products in surface water was faster than in synthetic solutions for both current densities. Furthermore, the 7.6 mA/cm^2^ current density resulted in faster degradation of oxidation products. Results obtained from this work are promising for practical applications because short reaction times and low current densities are needed for degradation of paracetamol and its oxidation products. These densities can be potentially supplied by photovoltaic cells.

## Introduction

Paracetamol is one of the most commonly prescribed pharmaceutical drugs (Yang *et al.*, 2008; Lourenção *et al.*, 2009; Solé *et al.*, 2010; Wu *et al.*, 2012), as it has been reported as safe for human usage in analgesic and antipyretic therapy (Xu *et al.*, 2008). It is considered one of the three most prescribed drugs, and is ranked among the 200 top prescriptions in the United States (Zhang *et al.*, 2008; Wu *et al.*, 2012).

**FIG. 1.**
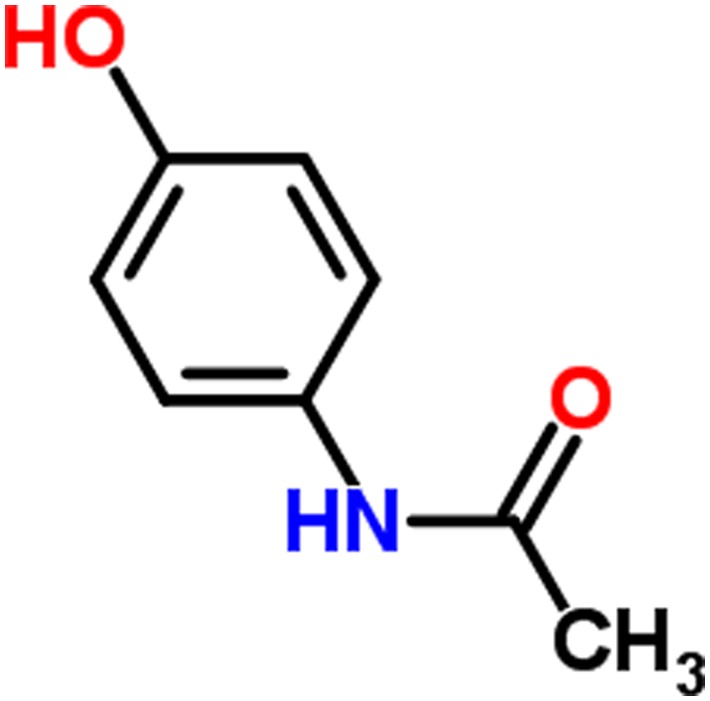
Functional groups of paracetamol.

Paracetamol has been found in aquatic ecosystems in the wild. This compound reaches the natural environment either through direct disposal of domestic drugs, discharges of feces/urine, or the inappropriate treatment of industrial effluents (Ganiyat, 2008; Yang *et al.*, 2008). The toxicity of paracetamol has been documented extensively in animals and humans (Jaeschke and Bajt, 2006; Brind, 2007; Xu *et al.*, 2008); nevertheless, there are a lack of studies focusing on marine/aquatic communities that are particularly vulnerable, especially filter feeders that can accumulate large amounts of pharmaceuticals within their bodies. Paracetamol contains three functional groups: the hydroxyl group (OH), the amide group (HN-CO-R), and the aromatic group (benzene ring), as shown in Fig. 1.

Wastewater treatment plant (WWTP) effluent is considered the main source of paracetamol in surface waters (Bolong *et al.*, 2009; Kasprzyk-Hordern *et al.*, 2009). The development of effective water treatment processes for degrading pharmaceuticals is a new challenge for the scientific community. Many studies have been conducted with a main purpose of finding an effective and viable method to degrade paracetamol. Among them, the following can be mentioned: electrocatalysis (Sánchez-Obrero *et al.*, 2011); photo-Fenton (Durán *et al.*, 2011); chemical (Hiremath *et al.*, 2006); sonolysis (Quesada-Peñate *et al.*, 2009); nonthermal plasma (Baloul *et al.*, 2016); photocatalysis (Kanakaraju *et al.*, 2014); reverse osmosis (Al-Rifai *et al.*, 2007); activated carbon (Rossner *et al.*, 2009); chlorination (Bedner and Maccrehan, 2006); and ozonation (Westerhoff *et al.*, 2005). In previous studies, only partial degradation of paracetamol was reported, between 14% and 88% (Basavaraju *et al.*, 2011; Durán *et al.*, 2011), instead of total mineralization.

Furthermore, in most studies, the intermediates/oxidation products were not degraded (Nematollahi *et al.*, 2009; Trovó *et al.*, 2012), the reaction times were too long (Edrees *et al.*, 2017), a high voltage was used (from 600 to 1,400 V), and a continuous injection of oxygen to generate H_2_O_2_ was required, resulting in very costly and ineffective methods. In recent decades, the development of technologies such as electrochemical oxidation processes have reached a promising stage of progress, providing versatility, energy efficiency, automation, environmental compatibility, and cost effectiveness, which with correct application are now used effectively in micropollutant removal (Anglada *et al.*, 2009).

However, an inconvenience of applying electrochemical oxidation to degrade emerging pollutants is the generation of intermediates/oxidation products (Zhao *et al.*, 2009). During the electro-oxidation of paracetamol conducted by Brillas *et al.* (2005) using a boron-doped diamond and platinum (Pt) anode at a pH range of 2.0–12.0, a release of $NH_4^ +$ and $NO_3^ -$ ions was observed in both anodes. In addition, hydroquinone and 1,4-benzoquinone were detected, while oxalic and oxamic acids were identified as ultimate carboxylic acids. Furthermore, p-aminophenol, p-nitrophenol, and NAPQI (N-acetyl-benzoquinone imine) have been detected in the advanced oxidation of paracetamol (Bedner and Maccrehan, 2006; Moctezuma *et al.*, 2012; Postigo and Richardson, 2014).

These by-products have the potential of having high levels of toxicity and/or could be even more bio-recalcitrant than the initial compound, making them harder to degrade using typical methods (Anglada *et al.*, 2009). For this reason, the degradation of not only paracetamol but also its oxidation products is of paramount importance. In this regard, López Zavala and Espinoza Estrada (2016) successfully oxidized paracetamol and its transformation products in synthetic solutions by using an electrochemical oxidation cell with stainless steel electrodes. However, due to constituents of surface water that could affect the effectiveness of paracetamol oxidation, it was necessary to evaluate the performance of the process when real surface water is treated.

Thus, in this study, electrochemical oxidation of paracetamol and its oxidation products in surface water was conducted under the optimum oxidation conditions obtained by López Zavala and Espinoza Estrada (2016) for synthetic solutions (pH 3 and direct current (DC) densities of 5.7 and 7.6 mA/cm^2^) and the results were compared with those of paracetamol synthetic solution oxidation. The results obtained in this study are promising for practical applications because not only the paracetamol was degraded but also its oxidation products; additionally, short reaction times and low DC densities were needed. Such DC densities can be potentially supplied by photovoltaic cells.

## Materials and Methods

### Chemicals and materials

Acetaminophen (4-acetamidophenol, 98%), potassium hydroxide (KOH), and sulfuric acid (${H_2}S{O_4}$), were obtained from Sigma-Aldrich (Toluca, Mexico). Hydrochloride acid (HCl) at 37% was purchased from Fermont (Mexico City, Mexico). Acetic acid was supplied by Fisher Scientific (Monterrey, Mexico). Methanol was provided by J.T. Baker (Center Valley, PA). Ultrapure water was obtained from a Milli-Q water purification system purchased from Bedford, MA. Surface water was collected from the dam “Rodrigo Gómez” located in Santiago, Nuevo León, Mexico, near the city of Monterrey.

### Experimental device

**FIG. 2.**
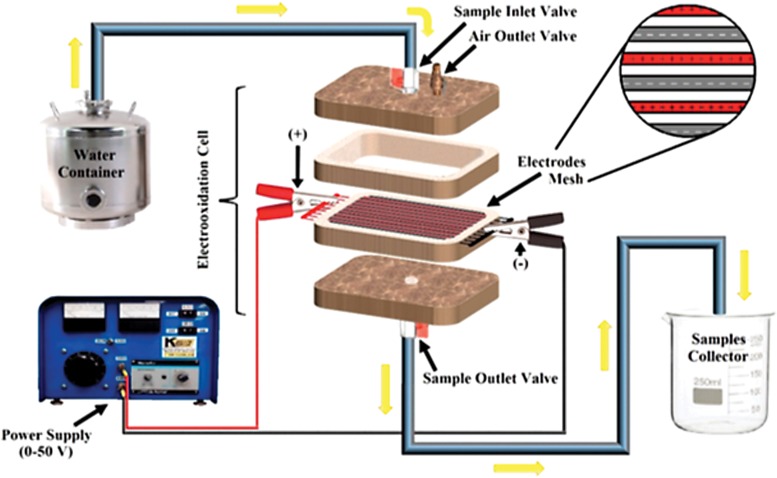
Electrochemical oxidation cell (López Zavala and Espinoza Estrada, 2016).

A home-built fiberglass electrochemical oxidation cell, reported by López Zavala and Espinoza Estrada (2016), was used to run all the tests, and it is presented in Fig. 2. The device has a total volume of 180 mL. In the third plate, a mesh of stainless steel electrodes is configured, and prepared based on a textile technique reported by Abidin *et al.* (2007). The dimensions of the mesh are 107 × 60 mm; it is integrated by 27 electrodes made of 24 American Wire Gauge (AWG) stainless steel wire with a 0.56 mm diameter, length of 107 mm, and spaced at 2.1 mm each. Thirteen electrodes functioned as cathodes and the other 14 worked as “active” anodes. The electrodes had a terminal connected to a DC power supplier (Kaselco from Seal Beach, CA) that can supply a voltage between 0 and 50 V, and a current intensity between 0 and 10 A. The electrochemical oxidation cell can be operated on either batch or continuous flow schemes; but in this study, batch operation was conducted.

### Characterization of surface water

Bulk characterization of surface water was conducted based on parameters such as pH, electrical conductivity (EC), chemical oxygen demand (COD), turbidity, total solids (TS), total dissolved solids (TDS), and paracetamol concentration. The pH and EC were determined by using a Thermo Scientific Orion 3-Star equipment (Thermo Fisher Scientific, Waltham, MA). Turbidity was measured based on the nephelometric method by using a Hach 2100 N Turbidimeter (USA Hach Company, Loveland, CO). COD, TS, and TDS were determined based on the standard methods for the analysis of water and wastewater (APHA, AWWA, and WEF, 2005). Paracetamol in surface water was analyzed by high-performance liquid chromatography using an Agilent 1200 HPLC-DAD system (Agilent Technologies, Santa Clara, CA).

### Degradation of paracetamol and its oxidation products

Solutions of 10 mg/L-paracetamol were prepared using surface water. The solution pH was adjusted from 6.8 to 3.0 by adding HCl. The adjustment of pH was conducted to run the experiments under the optimum oxidation conditions obtained by López Zavala and Espinoza Estrada (2016) for synthetic solutions (pH 3). Then, the solutions were treated electrochemically at DC densities of 5.7 mA/cm^2^ 6 V) and 7.6 mA/cm^2^ (12 V) and reaction times 1, 2.5, 5, 7.5, 10, 15, 20, 25, 30, 35, 40, 50, 60, 120, 240, 360, and 540 min. All tests were conducted by triplicate on batch configuration. For each reaction time, samples were taken and filtered using 0.45 μm polytetrafluoroethylene syringe filters. Then, they were analyzed by triplicate using an Agilent 1200 HPLC-DAD equipment (Agilent Technologies).

Separation of analytes was conducted by using a 150 × 4.6 mm reverse phase monomeric Zorbax C18 column with 5 μm diameter spherical particles (MAC-MOD Analytical, Wilmington, DE). Analysis of the paracetamol and its oxidation products was conducted based on the procedure reported by Alkharfy and Frye (2001). The mobile phase used was methanol and 1% acetic acid in ultrapure water (40/60/v/v) and the operating conditions of the equipment were flowrate of 1.0 mL/min, temperature 25°C, detection at 254 nm, and injection volume of 20 μL.

## Results and Discussion

### Surface water characterization

Raw surface water had low constituent load. Analysis by triplicate of raw surface water samples gave the following results: pH, 7.98; EC, 0.33 mS/cm; turbidity, 5.0 NTU; COD, 130.88 mg/L; TS, 113 mg/L; and TDS, 97 mg/L. Paracetamol traces in raw surface water were unable to be detected by high-performance liquid chromatography with diode-array detection (HPLC-DAD) at 254 nm. Once prepared, the 10 mg/L paracetamol solutions with surface water, the pH changed from 7.98 to 6.8.

### Degradation of paracetamol in surface water

**FIG. 3.**
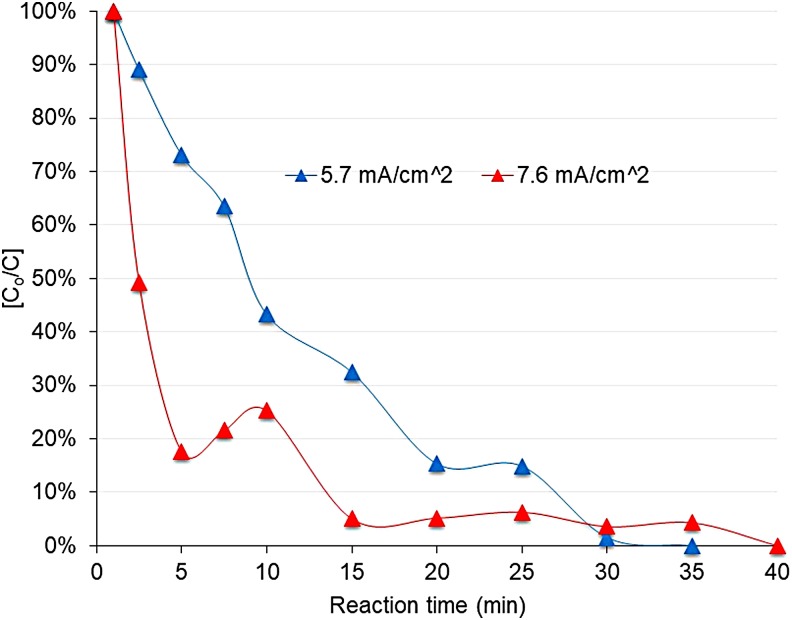
Decay of paracetamol at pH 3 and DC densities of 5.7 and 7.6 mA/cm^2^. The figure shows the average of three replicates. DC, direct current.

Paracetamol in surface water was totally degraded by using the electrochemical oxidation cell reported by López Zavala and Espinoza Estrada (2016). Figure 3 shows the decay of the paracetamol at pH 3 and DC densities of 5.7 mA/cm^2^ (6 V) and 7.6 mA/cm^2^. As seen in Fig. 3, degradation of the paracetamol was faster at 7.6 mA/cm^2^. At 5 min reaction time, the pharmaceutical had been degraded 82%, while at 5.7 mA/cm^2^, the degradation was ∼27%. Similarly, at 15 min reaction time, the decay of the paracetamol was 95% at 7.6 mA/cm^2^ and nearly 70% at 5.7 mA/cm^2^. Total degradation was achieved within 35 and 40 min for both current densities. López Zavala and Espinoza Estrada (2016) reported total degradation of paracetamol in synthetic solutions at similar reaction time for 5.7 mA/cm^2^ current density, while at 7.6 mA/cm^2^, total degradation occurred at 7.5 min reaction time, much shorter than in surface water.

Higher current density might enhance the deposition of sludge and hydroxides on the electrodes, which can inhibit the electrolysis process and interfere with the passage of current (Garcia-Segura *et al*. 2017), thus limiting the direct oxidation of the Cl^−^ ion at the anode. Less soluble chlorine formation might delay the oxidation of paracetamol in surface water. As mentioned above, surface water was conditioned with HCl to reach a pH 3; then, chloride ions are present due to the dissociation of the HCl into H^+^ and Cl^−^. Oxidation of paracetamol under these conditions is based on the direct oxidation of the Cl^−^ ion at the anodes to form soluble chlorine (Cl_2_), which is hydrolyzed and transformed into hypochlorous acid (HClO) according to Reactions (1) to (3):

\begin{align*}
2{ \rm{C}}{{ \rm{l}}^ - }  \to { \rm{ C}}{{ \rm{l}}_2} \;\left( {{ \rm{aq}}} \right)  + { \rm{ }}2{{ \rm{e}}^ - } , \tag{1}
\end{align*}

\begin{align*}
{ \rm{C}}{{ \rm{l}}_{2}} \ ({{\rm{aq}}}) + {{\rm{H}}_2}{ \rm{O}}
\rightleftarrows {\rm{HClO}} + { \rm{C}}{{ \rm{l}}^ -}  + {{
\rm{H}}^ + } , \tag{2}
\end{align*}

At pKa = 7.55, hypochlorous acid is in equilibrium with hypochlorite ion

\begin{align*}
{ \rm{HClO}} \rightleftarrows { \rm{Cl}}{{ \rm{O}}^ - }  + { \rm{
}}{{ \rm{H}}^ + } , \tag{3}
\end{align*}

Thus, degradation activity of “active” anodes (stainless steel electrodes) used in the electro-oxidation cell was remarkably enhanced by the presence of chloride ions in the surface water solution, as it was reported by López Zavala and Espinoza Estrada (2016) for synthetic paracetamol solutions.

### Degradation of paracetamol oxidation products

**FIG. 4.**
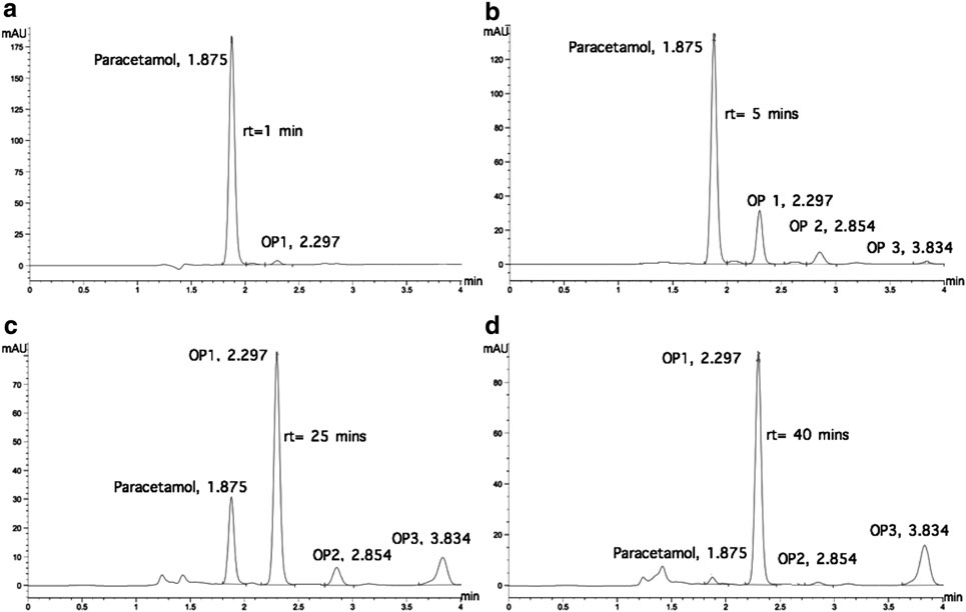
Chromatograms of paracetamol degradation and its oxidation products at different reaction times, with pH 3 and current density of 5.7 mA/cm^2^. **(a)** rt = 1 min; **(b)** rt = 5 min; **(c)** rt = 25 min; **(d)** rt = 40 min. OP refers to oxidation products.

**FIG. 5.**
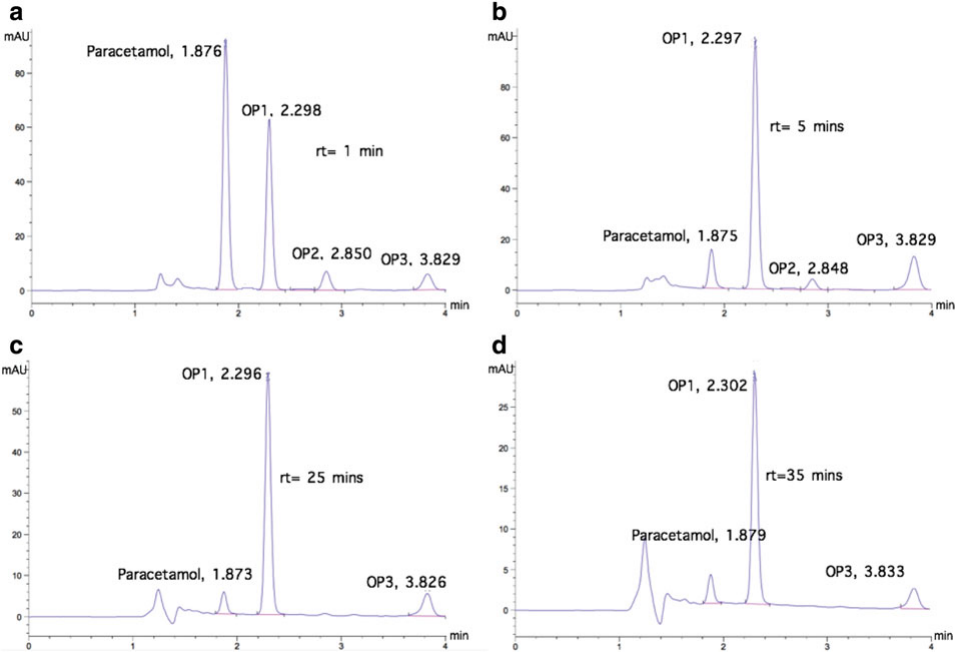
Chromatograms of paracetamol degradation and its oxidation products at different reaction times, with pH 3 and current density of 7.5 mA/cm^2^. **(a)** rt = 1 min; **(b)** rt = 5 min; **(c)** rt = 25 min; **(d)** rt = 35 min. OP refers to oxidation products.

Electro-oxidation of the paracetamol with active chlorine generated oxidation products. Therefore, these compounds had to be also degraded because of their potential toxicity. The chromatograms of Figs. 4 and 5 show the paracetamol degradation, the formation of oxidation products, and their degradation at DC densities 5.7 and 7.6 mA/cm^2^, respectively. Three oxidation products were detected and denoted as OP 1 (detention time 2.3 min), OP 2 (detention time 2.9 min), and OP 3 (detention time 3.8 min). These three oxidation products were the same detected by López Zavala and Espinoza Estrada (2016) in synthetic solutions. Their identification was not the scope of this work; nevertheless, they could correspond to those referred by the literature, such as 1,4- benzoquinone, p-aminophenol, p-nitrophenol, hydroquinone, and NAPQI (N-acetyl-benzoquinone imine) (Bedner and Maccrehan, 2006; Moctezuma *et al.*, 2012; Postigo and Richardson, 2014). As seen, the three oxidation products, observed by HPLC-DAD at 254 nm, were degraded successfully at both current densities.

**FIG. 6.**
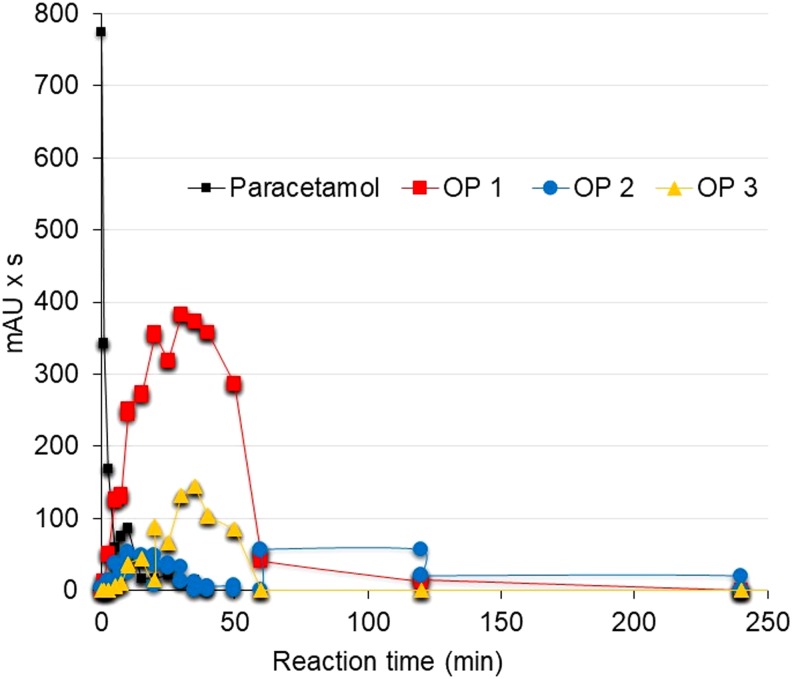
Degradation of oxidation products at pH 3 and current density 5.7 mA/cm^2^. The figure shows the average of three replicates.

**FIG. 7.**
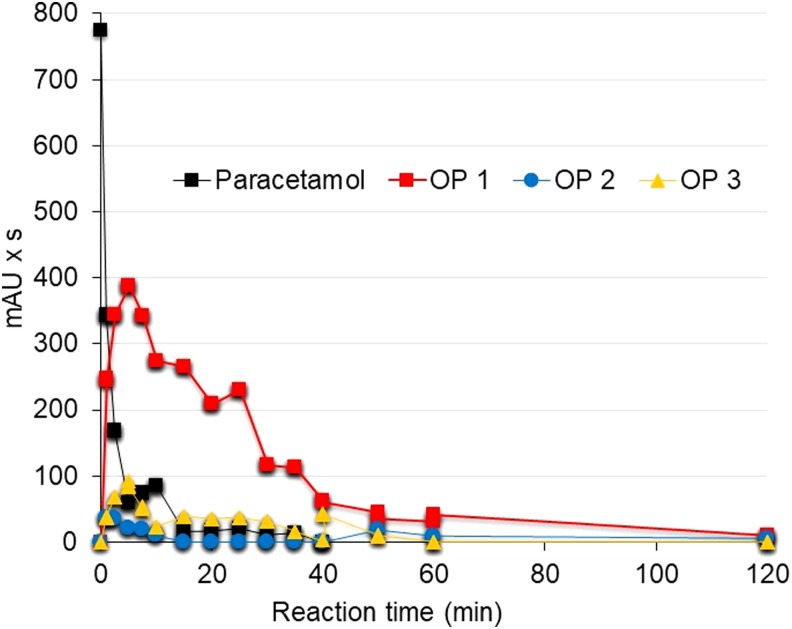
Degradation of oxidation products at pH 3 and current density 7.6 mA/cm^2^. The figure shows the average of three replicates.

At 5.7 mA/cm^2^, the oxidation products appeared immediately as the paracetamol is degraded and increased rapidly to reach the maximum detection when the pharmaceutical is already degraded (35–40 min); then, their detection decreased rapidly to 60 min detention time and, finally, they were not detected at 240 min reaction time (Fig. 6). At 7.6 mA/cm^2^, the oxidation products were also formed immediately as the paracetamol degradation started, but the detection peak was achieved faster, at only 5 min detention time; then, the detection decreased gradually to 60 min detention time and, finally, their detection was not observed at 120 min (Fig. 7). These reaction times are shorter than those values reported by López Zavala and Espinoza Estrada (2016) for synthetic solutions, 360 min at 5.7 mA/cm^2^ and 240 min at 7.6 mA/cm^2^.

In addition, in surface water, the decay of oxidation products occurred rapidly once the generation peaks were reached, while in the synthetic solution, their decay is steady downhill until they are no longer detected. The higher HCl concentration needed to acidify surface water, in comparison with the synthetic paracetamol solution, could result in a higher chloride concentration and consequently in a greater oxidation of the Cl^−^ ion at the anode. Thus, greater soluble chlorine formation could enhance the degradation of paracetamol oxidation products. These results are promising for practical applications because short reaction times and low current densities are required to achieve full degradation of paracetamol and its oxidation products.

Furthermore, current densities of 7.6 mA/cm^2^ (12 V) can be potentially supplied with photovoltaic cells. Thus, the approach presented in this study could become a potential sustainable technological alternative for degrading emergent contaminants. Certainly, more research must be conducted to (1) elucidate the chemical interactions/processes that are occurring between the paracetamol and its oxidation products with the surface water constituents, and (2) evaluate the degradation of other pharmaceuticals, hormones, personal care products (PCPs), and pesticides under low, neutral, and alkaline pH.

## Conclusion

Paracetamol solutions prepared with surface water were oxidized electrochemically at pH 3 and current densities of 5.7 mA/cm^2^ (6 V) and 7.6 mA/cm^2^ (12 V) using the stainless steel electrode cell reported by López Zavala and Espinoza Estrada (2016). The paracetamol was totally degraded in very short reaction times (35 to 40 min, respectively). In comparison with the results reported by López Zavala and Espinoza Estrada (2016) for electrochemical oxidation of paracetamol in synthetic solutions, the degradation of the pharmaceutical was faster in surface water than in the synthetic solution at 5.7 mA/cm^2^ (120 min reaction time in synthetic solution).

The higher HCl concentration needed to acidify surface water, in comparison with synthetic paracetamol solution, could result in a higher chloride concentration and consequently in a greater oxidation of the Cl^−^ ion at the anode. Thus, greater soluble chlorine formation could enhance the degradation rate of paracetamol. Nevertheless, at 7.6 mA/cm^2^, the total degradation of paracetamol in surface water was delayed up to 40 min, versus the 7.5 min in the synthetic solution. Deposition of sludge and hydroxides on the electrodes can inhibit the electrolysis process and interfere with the passage of current, thus limiting the direct oxidation of Cl^−^ that causes less formation of active chlorine species and consequently, longer reaction time.

Three oxidation products, observed by HPLC-DAD at 254 nm, in the paracetamol degradation were totally oxidized. In comparison with the synthetic solution, degradation of such oxidation products in surface water (240 and 120 min, respectively) was faster than in the synthetic solution (360 and 240 min, respectively) for 5.7 and 7.6 mA/cm^2^ current densities. The higher HCl concentration needed to acidify the surface water, in comparison with the synthetic paracetamol solution, could result in a higher chloride concentration and consequently in greater oxidation of the Cl^−^ ion at the anode. Thus, greater soluble chlorine formation could enhance the degradation of the paracetamol oxidation products. Higher current density resulted in faster degradation of the oxidation products.

Undoubtedly, more research is needed to elucidate the chemical interactions/processes that are enhancing the degradation rates of the oxidation products in surface water. However, the results obtained in this work are promising for practical applications because short reaction times and low current densities are needed. These current densities can be potentially supplied by photovoltaic cells, thus making the electrochemical oxidation process a potential sustainable technological alternative for degrading emergent contaminants.
